# Exposure to 50 Hz magnetic field modulates GABA_A_ currents in cerebellar granule neurons through an EP receptor-mediated PKC pathway

**DOI:** 10.1111/jcmm.12626

**Published:** 2015-07-14

**Authors:** Guang Yang, Zhen Ren, Yan-Ai Mei

**Affiliations:** a School of Life Sciences, Institute of Brain Science and State Key Laboratory of Medical Neurobiology, Fudan UniversityShanghai, China

**Keywords:** 50 Hz magnetic fields, GABA_A_ currents, EP receptors, PKC pathway, rat cerebellar granule neurons

## Abstract

Previous work from both our lab and others have indicated that exposure to 50 Hz magnetic fields (ELF-MF) was able to modify ion channel functions. However, very few studies have investigated the effects of MF on γ-aminobutyric acid (GABA) type A receptors (GABA_A_Rs) channel functioning, which are fundamental to overall neuronal excitability. Here, our major goal is to reveal the potential effects of ELF-MF on GABA_A_Rs activity in rat cerebellar granule neurons (CGNs). Our results indicated that exposing CGNs to 1 mT ELF-MF for 60 min. significantly increased GABA_A_R currents without modifying sensitivity to GABA. However, activation of PKA by db-cAMP failed to do so, but led to a slight decrease instead. On the other hand, PKC activation or inhibition by PMA or Bis and Docosahexaenoic acid (DHA) mimicked or eliminated the field-induced-increase of GABA_A_R currents. Western blot analysis indicated that the intracellular levels of phosphorylated PKC (pPKC) were significantly elevated after 60 min. of ELF-MF exposure, which was subsequently blocked by application of DHA or EP1 receptor-specific (prostaglandin E receptor 1) antagonist (SC19220), but not by EP2-EP4 receptor-specific antagonists. SC19220 also significantly inhibited the ELF-MF-induced elevation on GABA_A_R currents. Together, these data obviously demonstrated for the first time that neuronal GABA_A_ currents are significantly increased by ELF-MF exposure, and also suggest that these effects are mediated *via* an EP1 receptor-mediated PKC pathway. Future work will focus on a more comprehensive analysis of the physiological and/or pathological consequences of these effects.

## Introduction

Electromagnetic fields in the extremely low frequency (ELF) range is ubiquitously present in various environments in everyday life. The major sources of 50 Hz magnetic fields (ELF-MF) pertaining to the general public are in-house installations, household appliances and powerlines 1. A number of studies *in vitro* have noted that exposure to ELF-MF has multiple biological effects, including changes in gene expression, regulation of cell survival and promotion of cell differentiation 2,3. Recent studies have demonstrated that exposure to ELF-MF can produce higher order effects. For example, investigation by Salunke *et al*. (2014) indicated that long-term exposure to ELF-MF significantly increased anxiety without affecting locomotion, and there was a significant elevation of both GABA and glutamate levels in the hippocampus and hypothalamus of mice exposed 4. Furthermore, ELF-MF exposure can change dendritic spine density and morphology in the entorhinal cortical neurons, and had caused a long-lasting increase in the excitatory state of the neurons in the cortex and hippocampus 5,6. Although the effect of ELF-MFs on the activity of neuronal excitability controlling channels including Ca^2+^-active potassium channels and Na^+^ channels have previous investigated, very few studies have investigated the effects of ELF-MF on ligand-gated channels, particularly γ-aminobutyric acid (GABA) type A receptors (GABA_A_Rs).

It is well-known that inhibitory neurotransmission is largely mediated by GABA acting through GABA_A_Rs. These receptors are heteropentameric, ligand-gated chloride channels that belong to the Cys-loop ligand-gated ion channel superfamily 7. GABA_A_Rs are expressed ubiquitously in neurons along the entire neuraxis and their activity is important for animal development and neuronal differentiation 8,9. They are also critical for the structural and functional maturation of neurons 10–12. Moreover, deficits in GABA_A_R-mediated neurotransmission have been implicated in various pathophysiological disorders, such as anxiety, epilepsy and schizophrenia 13–15. Given the importance of GABA_A_Rs, studying the effects of ELF-MFs is required for an understanding of the possible causes of exposure-induced effects on learning and memory. However, there is currently little information as to whether ELF-MF can modulate GABA_A_R activity.

Cerebellar granule neurons (CGNs) constitute the largest, homogeneous neuronal population within the mammalian brain. Due to their postnatal generation and well-defined developmental pathway, CGNs have been established as an accurate *in vitro* model for studying neuronal development and maturation 16. Furthermore, *in vitro* CGNs cultures have also long been a model for studying GABA_A_ receptors 17,18 as well as a model for neuronal cell development and apoptosis 19–21. We have previously shown that exposure of CGNs to 10–60 min. of ELF-MF significantly increased Na_v_ currents (*I*_Na_) by 30–125% with a significant shift of their steady-state activation curve in both a time- and intensity-dependent manner 22. Since this phenomenon is similar to the effects seen with intracellular application of arachidonic acid (AA) and prostaglandin E_2_ (PGE_2_) on the *I*_Na_ in CGNs 23, a later mechanistic study revealed that the ELF-MF increase in neuronal *I*_Na_ occurs *via* a PKA-dependent pathway (cPLA2 → AA → PGE_2_ → EP receptors → PKA).

Therefore, the objective of this study was (*i*) to determine whether exposure to ELF-MF affected GABA_A_ receptor currents and (*ii*) whether a PKA-dependent mechanism might be involved. Our results demonstrate for the first time that GABA_A_ receptor currents are significantly increased by ELF-MF exposure *via* an EP receptor-mediated PKC signalling pathway.

## Materials and methods

### Ethics statement

This study was carried out in strict accordance with the Guide for the Care and Use of Laboratory Animals of the National Institutes of Health. The protocol was approved by the Committee on the Ethics of Animal Experiments of Fudan University (Permit Number: 20090614-001). All surgeries were performed under sodium pentobarbital anaesthesia and all efforts were made to minimize animal suffering.

### Cell culture

Cells were derived from the cerebellum of 7–8-day-old Sprague–Dawley rat pups as previously described 24. Isolated cells were plated onto 35 mm diameter Petri dishes coated with poly-l-lysine (10 μg/ml) at a density of 10^6^ cells/ml. Cultured cells were incubated at 37°C under 5% CO_2_ in DMEM supplemented with 10% foetal calf serum, glutamine (5 mM), insulin (5 μg/ml), KCl (25 mM) and 1% antibiotic–antimycotic solution (25 μg Streptomycin, 10,000 μg Amphotericin B, 10,000 UI Penicillin). All experiments were carried out using primary CGNs after 5–7 days in culture.

### ELF-MF exposure system

We used the same system (I-ONE, Shanghai, China) for magnetic field exposure of cerebellar GCs as has been used in previous studies, with some revisions 25–28. Briefly, a 50 Hz magnetic field was generated by a pair of horizontal Helmholtz coils (20 cm in height, and 20 cm in radius, each plate consists of 150 turns of copper wire) placed parallel to each other. The coils were powered by a generator system, which consists with a signal generator and an amplifier, that produced the input voltage of the pulse, and resulting magnetic flux densities could be regulated within the range 0–1.0 mT. Both the ELF-MF frequency and flux density were monitored by a MF sensor that was connected to a digital multimeter. The geometry of the system assured a uniform field in the area of a central cylinder (10 cm in height and 6 cm in radius) for the exposed cultured cells. The surfaces of the culture plates were perpendicular to the force lines of the alternating magnetic field in the solenoid. Air and culture medium temperatures were continuously monitored for the duration of all experiments 22. The incubator was keep closed all throughout the ELF-MF or non-MF experiments to make sure that the conditions remained stable. Non-MF groups (sham) were incubated in the same incubator in which the conditions were the same as for the exposed groups, but MF exposure system was off.

### GABA_A_R current recordings

Whole-cell currents from granule neurons were recorded with a patch-clamp technique. Prior to GABA_A_R current recordings, the culture medium was replaced with a bath solution containing the following: NaCl 145 mM, KCl 2.5 mM, HEPES (4-(2-hydroxyethyl)-1-piperazineethanesulfonic acid) 10 mM, MgCl_2_ 1 mM and glucose 10 mM (pH adjusted to 7.4 with NaOH). Soft-glass pipettes (BR749321 BRAND® micro haematocrit capillary, Sigma-Aldrich, St. Louis, MO, USA) were filled with an internal solution containing the following: KCl 145 mM, HEPES 10 mM, CaCl_2_ 1 mM, MgCl_2_ 1 mM, ethylene glycol tetraacetic acid (EGTA) 10 mM and ATP 1 mM (pH adjusted to 7.2 with KOH). The pipette resistance was 5–7 MΩ after filling with the internal solution. The recordings were performed at 23–25°C. GABA_A_ currents were recorded while the membrane potential was held at −70 mV. 100 μM GABA was given for 3 sec. using a gravity perfusion system to induce an inward Cl^−^ current. There was a 40 sec. interval between each GABA perfusion 29,30. In the protocol to study the concentration-response relationship of GABAA receptors, we used a 20 sec. interval between GABA applications instead of the 40 sec. interval. All currents were recorded using an Axopatch 700B amplifier (Axon Instruments, Foster City, CA, USA) operated in voltage-clamp mode using a computer connected to the recording equipment *via* a Digidata 1440A analog-to-digital (A/D) interface. Current was digitally sampled at 100 μsec. (10 kHz). Current signals were filtered by a 1 kHz, three-pole Bessel filter. Data acquisition and analysis were performed with pClamp 10.2 software (Axon Instruments) and/or Origin8.0 (Microcal Analysis Software, Northampton, MA, USA).

### Western blotting

The cells were lysed in HEPES-NP40 lysis buffer (20 mM HEPES, 150 mM NaCl, 0.5% NP-40, 10% glycerol, 2 mM ethylenediaminetetraacetic acid, 100 μM Na_3_VO_4_, 50 mM NaF, pH 7.5 and 1% proteinase inhibitor cocktail) on ice for 30 min. After centrifugation, the supernatant was mixed with 2× SDS loading buffer and boiled for 5 min. Proteins were separated on a 10% polyacrylamide gel, transferred to polyvinylidene difluoride membranes (Millipore, Billerica, MA, USA), blocked with 10% non-fat milk and incubated at 4°C overnight with either a rabbit polyclonal antibody against phosphorylated PKC (pPKC) PAN (#9371; Cell Signaling Technology, Beverly, MA, USA) or a mouse monoclonal antibody against Glyceraldehyde 3-phosphate dehydrogenase (GAPDH) (KC-5G4; KangChen Bio-tech, Shanghai, China). After extensive washing with TBST (Tris-Buffered Saline with Tween-20), the membrane was incubated with horseradish peroxidase-conjugated anti-mouse or anti-rabbit IgG (1:10,000; KangChen Bio-Tech) for 2 hrs at room temperature. Chemiluminescent signals were generated using a SuperSignal West Pico trial kit (Thermo Fisher Scientific Inc., Waltham, MA, USA) and detected using a ChemiDoc XRS System (Bio-Rad Laboratories Inc., Hercules, CA, USA). All measured protein bands were normalized to GAPDH and sham/GAPDH was set to 1.0.

### Chemicals

AH23848 hemicalcium salt (A8227), AH6809 (A1221), bisindolylmaleimide (Bis, B3931), cis-4,7,10,13,16,19-Docosahexaenoic acid (DHA, D2534), dibutyryl cyclic AMP (db-cAMP, D0627), Ethylene glycol-bis(2-aminoethylether)-N,N,N?,N?-tetraacetic acid (EGTA, E4378), 4-(2-Hydroxyethyl)piperazine-1-ethanesulfonic acid (HEPES, H3375), Insulin (I4011), Phorbol 12-myristate 13-acetate (PMA, P8139), poly-l-lysine (P2636), Prostaglandin E2 (PGE_2_, P5640) and SC 19220 (S3065) were all purchased from Sigma-Aldrich (St. Louis, MO, USA). L-798, 106 (cat. no. 3342) was from Tocris Bioscience (Bristol, UK). DMEM (12100-046), Foetal calf serum (10099-141), and the antibiotic–antimycotic (15240-062) solution were all purchased from Gibco Life Technologies (Grand Island, NY, USA).

### Statistical analysis

Statistical analysis was performed using a Student’s *t*-test with either a non-paired or paired comparison, as relevant. Values are given as the means ± SEM, with n representing the number of cells tested. A value of *P* < 0.05 was considered a statistically significant difference between groups. When multiple comparisons were made, the data were analysed using a one-way anova followed by *post* hoc analysis with Tukey and Fisher LSD tests for samples of more than two. All analyses were performed using Origin Pro software (OriginLab Corporation, Northampton, MA, USA).

## Results

### ELF-MF exposure increased GABA_A_ receptor currents without modifying their sensitivity to GABA

**Figure 1:**
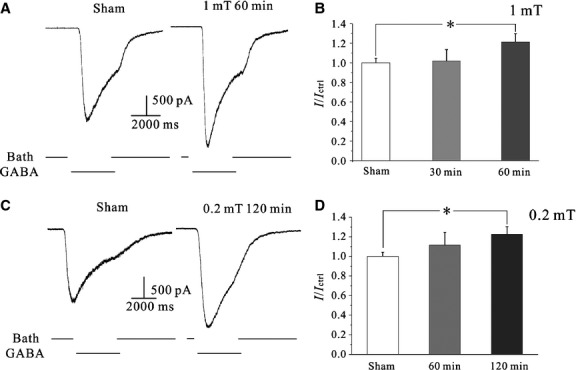
Effects of 50 Hz ELF-MF on CGN GABA_A_ receptor currents. (A) Current traces for the effects of ELF-MF (1 mT for 1 hr) on GABA_A_ receptor currents. (B) Statistical analysis of the time-dependent effects of ELF-MF exposure on GABA_A_ receptor current amplitudes. Data are means ± SEM. *P < 0.05 by one-way anova for two groups connected by a straight line. (C) Representative current traces for the effects of MF exposure (0.2 mT for 2 hr) on GABA_A_ receptor currents. (D) Statistical analysis of the time-dependent effects of ELF-MF exposure on GABA_A_ currents. Data are means ± SEM. *P < 0.05 by one-way anova for two groups connected by a straight line.

To investigate whether ELF-MF exposure modified GABA_A_ receptor current amplitudes in CGNs, CGNs were exposed to ELF-MF (1 mT) for 60 min., the amplitude of the GABA_A_ currents increased by approximately 21.5% ± 8.4% as compared to cells that had received no ELF-MF exposure (Fig. 1A, *n* = 10, *P* < 0.05). When ELF-MF exposure was shorter than 60 min., GABA_A_R currents were not significantly increased (Fig. 1B).

We also tested the effects of low-intensity ELF-MF (0.2 mT) on GABA_A_ currents. As shown in Figure 1C, when CGNs were exposed to 0.2 mT ELF-MF, a longer exposure time was needed to induce an increase in GABA_A_R currents. Moreover, the amplitude of GABA_A_R currents only increased by 11.5% ± 12.9% (*n* = 7, *P* > 0.05) when CGNs were exposed to ELF-MF (0.2 mT) for 1 hr. When the ELF-MF (0.2 mT) exposure time was increased to 2 hrs, the GABA_A_R current amplitude was increased by 22.8% ± 7.6% (Fig. 1D, *n* = 14, *P* < 0.05). However, the mean capacitance of the recorded cells in the sham group (8.85 ± 0.55 pF, *n* = 15) and for the ELF-MF treatment group (9.47 ± 0.41 pF, *n* = 14) showed no significant difference (*P* > 0.05). This similarity in capacitance indicates that the ELF-MF-induced increase in current amplitude was not due to differences or abnormalities in cell morphology.

Previous work has indicated that long-term cellular ELF-MF exposure may alter levels of protein expression 31, leading to potential difficulties in identifying the primary factor involved in our observed ELF-MF-induced increases in GABA_A_R currents. Thus, we chose to focus on the mechanism by which a relatively short-term exposure to a 1 mT ELF-MF (60 min.) induces increases in GABA_A_R currents.

**Figure 2:**
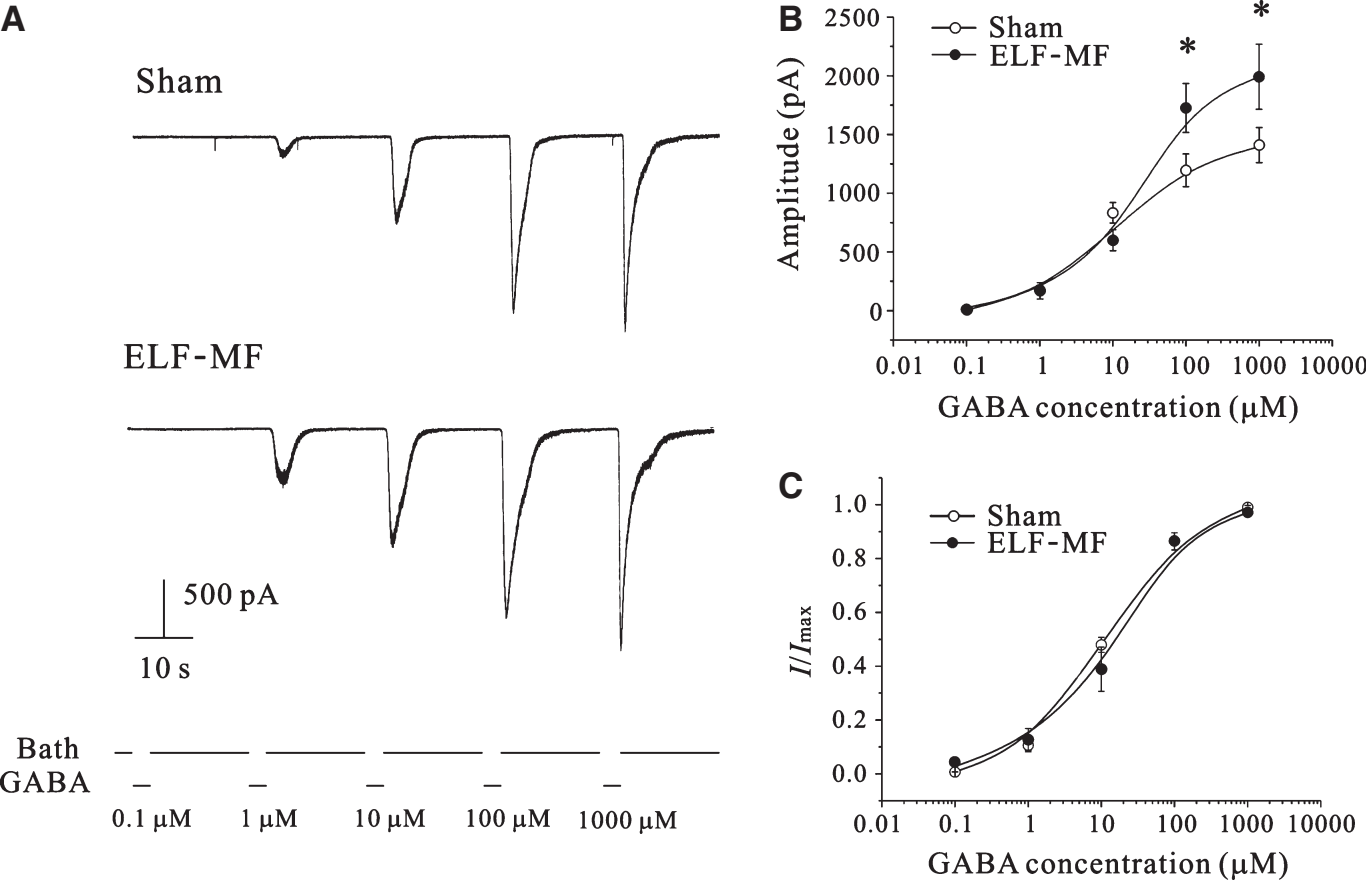
Effects of ELF-MF on GABA sensitivity of GABA_A_ receptor. (A) GABA_A_ currents elicited by different concentrations of GABA applied to the cells with a gravity perfusion system in either sham or MF exposure groups (1 mT for 1 hr). (B) Curve of GABA_A_ receptor current amplitude as a function of GABA concentration with control (sham exposure) or ELF-MF exposure. (C) Plot of the normalized current amplitude as a function of GABA concentration with the treatment of sham or ELF-MF exposure; Data points were processed by Hill regressions in Origin 8.0. Data are means ± SEM. *P < 0.05 by two-sample *t*-test for two groups connected by a straight line.

The increase in GABA_A_ receptor current amplitude could also be due to an increase in the sensitivity of receptors to GABA. To test this hypothesis, we applied the GABA concentration-dependent experiment for CGNs with or without ELF-MF exposure. Currents were recorded while the membrane potential was held at −70 mV. Different concentrations of GABA (100 nM to 1 mM, beginning with the lower range) were given to induce GABA receptor current in CGNs with 20 sec. intervals between each concentration (Fig. 2A). The data were then fitted with the Hill equation: *I* = *I*_max_/(1 + (EC_50_/[GABA])^*n*_H_), where the GABA-induced current *I* is a function of the GABA concentration, EC_50_ is the GABA concentration required for inducing a half-maximal current, *n*_H_ is the Hill coefficient, and *I*_max_ is the maximum current. The maximum current was then used to normalize the concentration-response curve for each individual trace. The average of the normalized currents for each GABA concentration was used to plot the data. Our results showed that the GABA_A_ receptor current was significantly increased with ELF-MF exposure (Fig. 2B). However, the GABA_A_ receptor concentration-response relationship was not significantly changed by ELF-MF exposure when compared to the non-ELF-MF exposed controls (Fig. 2C). Fitting the normalized current amplitude as a function of GABA concentration using the Hill formula indicates that the Hill regression parameters of sham or ELF-MF exposure have no significant difference (Table 1). Collectively, these results indicate that the ELF-MF-induced increases of GABA_A_ receptor currents is not due to an increase in receptor sensitivity to GABA.

**Table 1:** Hill regression parameters of Concentration- response relationship of GABA_A_ current with sham or ELF-MF exposure (*n* = 6–7)

	Sham	ELF-MF
Normalized *I*_max_	1.00 ± 0.01	1.02 ± 0.05
EC_50_	11.80 ± 2.04	15.73 ± 3.76
*n*_H_	1.04 ± 0.04	0.82 ± 0.13
*K*_d_ = EC_50_^*n*_H_	13.02	9.67

### ELF-MF exposure increased GABA_A_ receptor currents by activation of a PKC-dependent pathway

**Figure 3:**
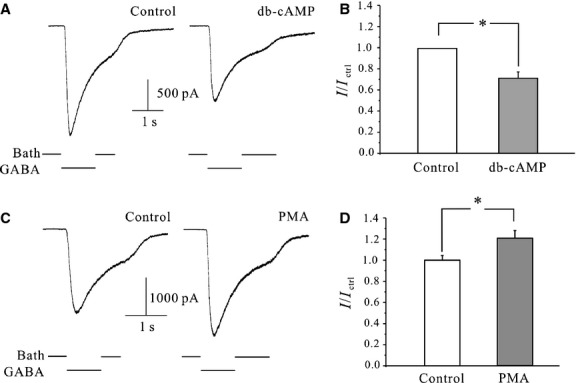
Effects of PKA or PKC pathway on CGNs GABA_A_ receptor currents. (A) Control currents and those following the application of 20 μM db-cAMP for 1 hr. (B) Statistical analysis of the effects of 1-hr application of db-cAMP on GABA_A_ currents. (C) Control currents and currents following the application of 100 nM PMA for 1 hr. (D) Statistical analysis of the effects of PKC activation on GABA_A_ currents. Data are means ± SEM. *P < 0.05 by two-sample *t*-test for two groups connected by a straight line.

Previous studies have indicated that GABA_A_ receptor activity can be modulated by the activation of protein kinase A 32. As such, db-cAMP (a cAMP analogue) was added to the bath solution and CGNs were then incubated for 1 hr to elucidate whether a PKA-dependent pathway was involved in effects of ELF-MF exposure. However, 20 μM db-cAMP was unable to mimic ELF-MF exposure-induced increases in GABA_A_R currents. Interestingly, it reduced the current amplitude by 28.6 ± 6.1% (*n* = 8; Fig. 3A), suggesting that a PKA pathway was not involved in the effects of ELF-MF on GABA_A_-R currents increase. Since a previous study had reported that GABA_A_R currents were modified in a PKC-dependent manner 33,34, we thus identified whether a PKC pathway was involved in the ELF-MF exposure-induced increases of GABA_A_R currents. Incubating CGNs with 100 nM PMA (a PKC activator) in the bath solution for 1 hr was able to partly mimic the enhancement effects of ELF-MF exposure on GABA_A_R currents (Fig. 3B), increasing GABA_A_R current amplitude by 20.4 ± 7.2% (*n* = 28, *P* < 0.05).

**Figure 4:**
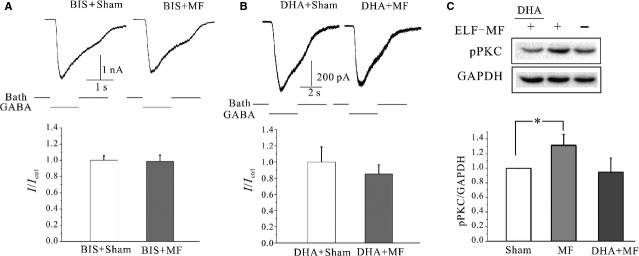
Effects of activation of PKC pathway on ELF-MF-induced increases of GABA_A_ currents. (A) Current traces in the presence of 10 μM Bisindolylmaleimide (Bis) in sham or 1 mT 1 hr MF exposure-treated groups (upper panel), and statistical analysis of the effects of Bis in ELF-MF-induced increases of GABA_A_ currents (lower panel). (B) Current traces in the present of 10 μM DHA in sham or ELF-MF exposure-treated groups (upper panel), and statistical analysis of the effects of DHA on ELF-MF-induced increases of GABA_A_ currents (lower panel). (C) Western blot and statistical analysis of the effects of DHA on pPKC levels in sham or ELF-MF exposure-treated groups. Data are means ± SEM. *P < 0.05 by two-sample *t*-test for two groups connected by a straight line.

Moreover, the effects of ELF-MF exposure-induced increase of GABA_A_R currents were attenuated upon application of both Bis and DHA, two PKC inhibitors (Fig. 4A and B). ELF-MF exposure slightly decreased GABA_A_ receptor currents by 9.6 ± 7.3% in the present of Bis (*P* > 0.05, when compared to Bis with sham, *n* = 15). In the present of DHA, ELF-MF exposure did not result in an increase of GABA_A_ receptor currents, but rather a slight decrease in current amplitude by 15.0 ± 11.2% (*P* > 0.05, when compared to DHA with sham). Taken together, these data suggest that a PKC pathway may be involved in the increases in current amplitude seen upon ELF-MF exposure.

To confirm the involvement of a PKC-dependent pathway in the ELF-MF exposed increases of GABA_A_ receptor currents, we used Western blotting in conjunction with a phospho-specific antibody to measure levels of pPKC in response to ELF-MF exposure. As shown in Figure 4C, there was a significant increase in pPKC levels (30.8 ± 14.9%, *P* < 0.05) after a 1 hr exposure of CGNs to ELF-MF. Moreover, the inhibition of PKC activity by DHA effectively eliminated the ELF-MF exposure-induced increases of pPKC (Fig. 4C).

### EP1 receptor activation was associated with ELF-MF exposure increases in GABA_A_ receptor currents

**Figure 5:**
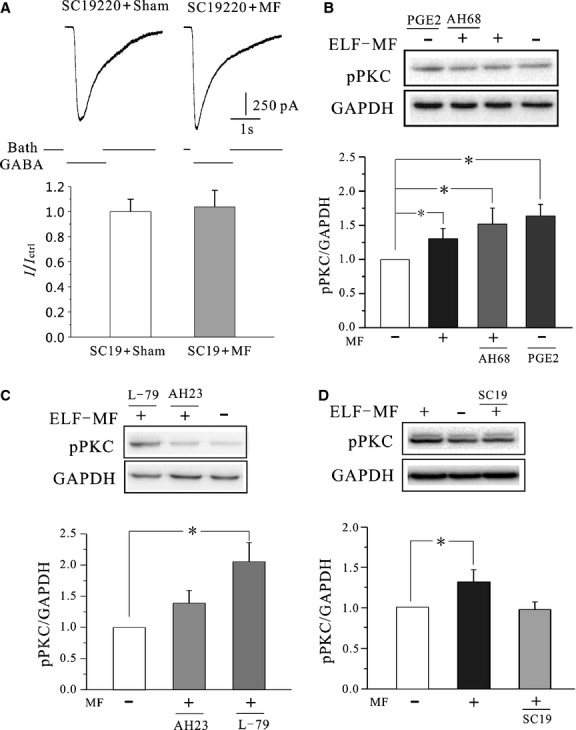
Effects of EP1 antagonists on ELF-MF-induced increases of GABA_A_ currents. (A) Current traces in the present of 20 μM EP1 receptor antagonist SC19220 with or without 1 mT MF exposure for 1 hr (upper panel), and statistical analysis of the effects of SC19220 on ELF-MF-induced increases of GABA_A_ currents by two-sample *t*-test (lower panel). (B–D) Western blot and statistical analysis of the effects of AH6809 (AH68), AH23848 (AH23), L-798,106, and SC19220 (SC19) on pPKC levels of CGNs with or without ELF-MF exposure. Data are means ±SEM. *P < 0.05 by one-way anova for two groups connected by a straight line.

**Figure 6:**
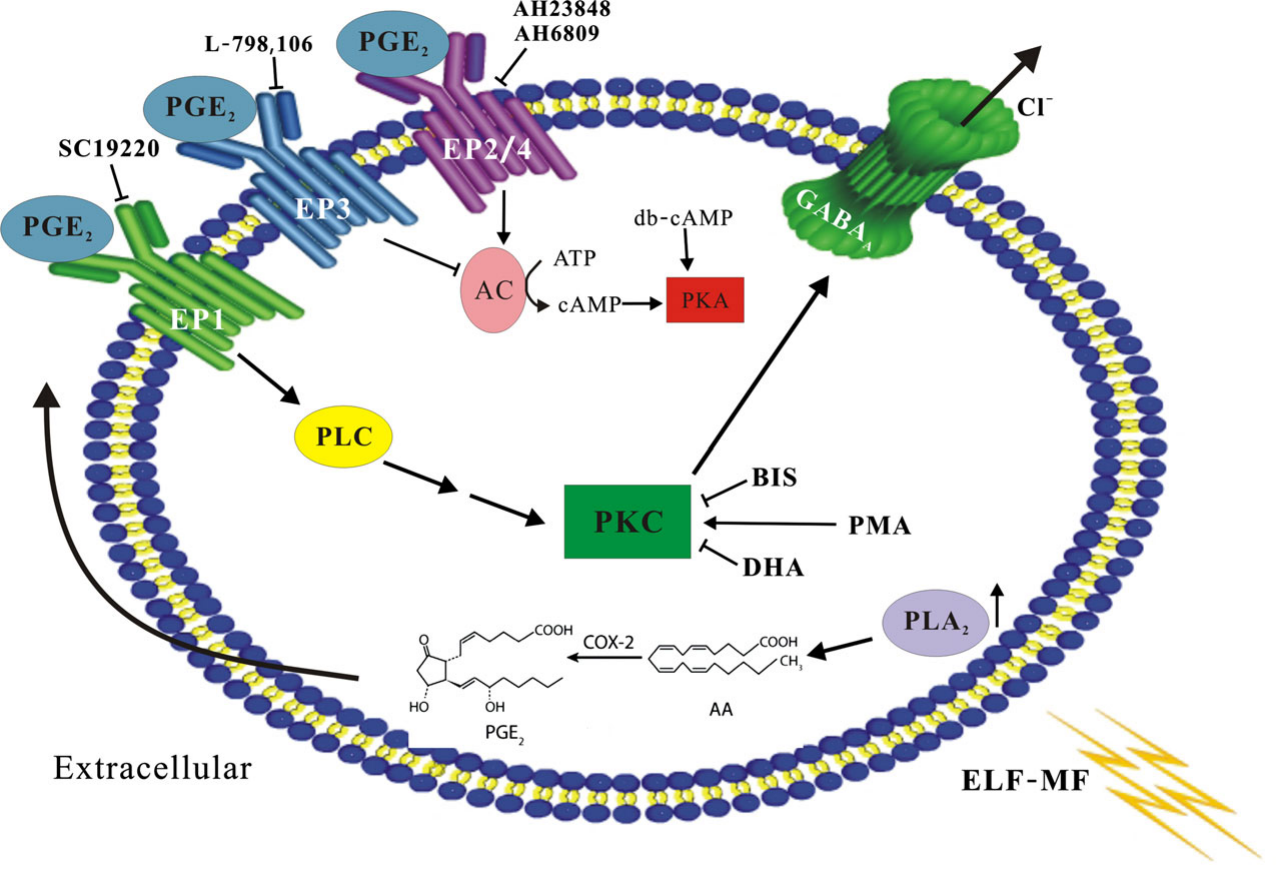
A proposed model depicting the mechanisms that are likely to be involved in the modulation of GABA_A_ receptors currents by ELF-MF exposure in CGNs. ELF-EMF active cPLA2 and up-regulated AA and PGE2, which can act in an autocrine or paracrine manner to activate EP receptors. Ligand binding of EP1 is associated with PKC activation and consequently modulates GABA_A_ receptors currents.

Our previous study indicated that there was an increase in intracellular AA and PGE_2_ levels following ELF-MF exposure in CGNs. Furthermore, that ELF-MF exposure significantly increased neuronal *I*_Na_ through PGE_2_ receptor activation 22. We therefore used Prostanoid EP receptor antagonists to examine whether EP receptors mediated the observed increases in GABA_A_ current and pPKC levels as a result of ELF-MF exposure. In the presence of 20 μM SC19220 (an EP1 receptor-specific antagonist) the effects of ELF-MF increase of GABA_A_ receptor currents were reduced to 3.7 ± 13.2% (Fig. 5A, *n* = 17, *P* > 0.05 when compared to SC19220 with sham). Similarly, SC19220 also eliminated the effects of ELF-MF on pPKC levels. In the presence of SC19220, ELF-MF mildly increased pPKC levels by 0.4 ± 9.7% (Fig. 5D, *n* = 5, *P* > 0.05 when compared to sham). Importantly, AH23848 (an EP2 receptor specific-antagonist), L-798, 106 (an EP3 receptor specific-antagonist), and AH6809 (an EP4 specific-antagonist) were all unable to reduce or ablate the effects of MF-induced increases in pPKC levels (Fig. 5B and C, *n* = 10). Taken together, results suggest that a PKC pathway activated by EP1 receptors mediates the MF-induced increases in GABA_A_R current (Fig. 6).

## Discussion

Although ELF-MF exposure has been previously reported to modulate the activity of voltage-gated ion channels, including sodium and calcium channels 22,35, few studies to date measured the effects of MF exposure on neuronal, ligand-gated ion channels. For the first time, we report here that ELF-MF exposure enhances GABA_A_ receptor currents in cerebellar GCs. In particular, neuronal exposure to ELF-MF influences EP receptor-mediated activity of a PKC-dependent pathway and accounts for the induction of GABA_A_ receptor currents.

Our previous study revealed that intracellular AA and PGE_2_ levels were increased following ELF-MF exposure by enhancing cPLA_2_ activity in cerebellar GCs. Increased PGE_2_ activated a PKA pathway *via* EP receptors, which then enhanced neuronal *I*_Na_.^22^ In this study, our results indicated that ELF-MF-induced increases of PGE_2_ not only modified neuronal *I*_Na_, but also modified neuronal GABA_A_ receptor currents. Interestingly, we noted that although this ELF-MF exposure-induced response is modulated by induction of PGE_2_, the PKA signalling pathway that induced enhancement of *I*_Na_ did not associate with ELF-MF-induced increase of GABA_A_ currents. Although PKA has been previously shown to be a modulator of GABA_A_ receptors, thereby enhancing or reducing the function of neuronal GABA_A_ receptors by acute reduction in channel opening frequency or chlorine increases in gene expression 32,36. However, the activation of a PKC signalling pathway was shown to underlie the effects seen in our study. This might result from the differential activation of PGE_2_ receptor. A higher level of PGE_2_ might be produced by ELF-MF exposure since our previous study identified that all of four types EP receptors are expressed in CGNs 23.

Numerous studies have indicated that PGE_2_ is mediated by the family of EP receptors, which consists of four isoforms: EP1-EP4 37,38. When activated, EP2 and EP4 receptors increase the concentration of intracellular cAMP 39,40, but EP3 activation resulted in a decrease of intracellular cAMP levels 41. However, EP1 receptor activation coupled to increase phospholipase C (PLC), which mediates activation of protein kinase (PKC) and subsequent elevation of cytosolic free calcium 42. Although our previous study identified that all four types of EP receptors were expressed in CGNs 23, pharmacological experiments with both EP receptor-specific antagonists and agonists indicated that ELF-MF exposure and PGE_2_-induced increases of GABA_A_ currents was mediated solely by activation of the EP1 receptor. This explains why it is the PKC rather than the PKA pathway that is associated with ELF-MF exposure-induced increases in GABA_A_ receptor currents.

Although our previous study suggested that ELF-MF exposure increased PKA activity, our study here showed that activating PKA alone should induce an inhibitory effect on GABA_A_ receptor currents. This is because incubation of CGNs with db-cAMP reduced the amplitude of GABA_A_ receptor currents. However, ELF-MF exposure-induced PKA activity did not seem to offset the PKC-driven effects induced by ELF-MF exposure on GABA_A_ currents since both the ELF-MF driven increases in percentage of current amplitude and PMA stimulation were similar. It is likely that PKA activity induced by ELF-MF exposure occurred prior to PKC activation. This is because immunoblot assays of intracellular levels of pPKA indicated a significant increase in ELF-MF exposure-induced levels of phospho-PKA (10–30 min.) 22. The time difference among PKA, PKC or other kinase signal pathway activation raises the issue of the complexity of the biological effects induced by ELF-MF exposure. This, along with differences in experimental conditions and/or intensity and duration of MF exposure, partly explains why the reported results involved in the neuronal effects of ELF-MF in various organisms are variable and/or contradictory.

Phosphorylation of the intracellular domains between transmembrane domain 3 and 4 of the GABA_A_ receptor β and γ subunits by serine/threonine and tyrosine kinases has been shown to alter receptor function. This is either by a direct effect on receptor properties, such as the probability of channel opening or desensitization, or by regulating trafficking of the receptor to and from the cell surface 43,44. Previous immunocytochemical work have shown the expression of all three βsubunit types (β1, β2 and β3) in the granule cell layer 45, thus PKC might modify GABA_A_ receptor by phosphorylation of this subunit. The PGE_2_/EP1/PKC-mediated increases of GABA_A_ receptor currents occurred after a 60 min. ELF-MF exposure. However, ELF-MF exposure failed to induce changes in receptor sensitivity to GABA, thus the mechanism of upregulation might be independent of the transcription or translation of GABA_A_ receptor proteins. An additional possibility is that it is involved in the regulation of GABA_A_ receptor trafficking to the membrane.

Previous studies have shown that the modified effects of PKC activation on GABA_A_ receptors is diverse and appears to be dependent on subunit composition 34,46. Currently, there is evidence suggesting that PKC upregulates both GABA_A_ receptor cell-surface expression and their resulting surface stability 47. However, in cultured cortical neurons, PKC activity leads to a decrease in cell-surface GABA_A_ receptors and associated currents by phosphorylation of other proteins within the endocytic cascade 33,48. Furthermore, cell surface expression and postsynaptic accumulation of GABA_A_ receptors is a complex process, including subunit gene expression, assembly of subunits into receptors, as well as exocytosis, endocytic recycling, diffusion dynamics and the degradation of GABA_A_ receptors 49. As we have no evidence here to indicate whether ELF-MF exposure-induced and PGE_2_/EP1/PKC-mediated upregulation of GABA_A_ receptors are associated with its trafficking to the membrane, further research will be required to test this possibility and its underlying mechanism.

The neuronal effects of ELF-MF have been extensively studied in various organisms 50,51. Although the reported results are variable and/or contradictory, this is due in part to differences in experimental conditions and in the flux density and/or duration of MF exposure. However, MF has recently been reported to modulate neuronal excitability and neurogenesis 51–53. Coincidently, GABA_A_ receptor activity not only serves to regulate the excitability of neural circuits, but also plays a role in neuronal development and differentiation 8,9. Thus, our *in vitro* findings on the effects of ELF-MF exposure on GABA_A_ receptors may provide evidence and mechanistic insight as to the effects of MF on neuronal excitation and neurogenesis in the central nervous system (CNS). Moreover, deficits in GABA_A_R-mediated neurotransmission are known to associate with pathophysiological disorders, including anxiety disorders, epilepsy and schizophrenia 13–15. Therefore, further exploration is required to comprehensively analyse the physiological and/or pathological effects of ELF-MF exposure on GABA_A_ receptors. Moreover, whether ELF-MF-induced enhancement of GABA_A_ receptors may be relevant for (*i*) the treatment of brain disorders associated with deficits in GABA_A_ receptor functioning or (*ii*) as a potential therapeutic approach for disorders associated with neurogenesis. In addition, we did not address the mechanism by which ELF-MF triggered the bio-effect pathway (cPLA2 → AA → PGE_2_ → EP receptors) in the present study. Combining our current study with our previous data, we speculate that magnetic field may induce an electrophoretic effect of cell surface protein molecules and change the charge distribution of the cell membrane surface, which then enhances cPLA_2_ activity and increased intracellular AA and PGE_2_ levels in CGNs. Finally, since 1 mT of exposure is not encountered in the daily lives of the general public and seldom in occupational settings, the significance of our work is more applicable to biological mechanisms than population health risk assessments.
